# Examination of the TPMT and NUDT15*3 Variants to Predict the Response to Thiopurines in an Italian Cohort of Patients with Inflammatory Bowel Disease

**DOI:** 10.3390/ijms26167860

**Published:** 2025-08-14

**Authors:** Francesca Tavano, Orazio Palmieri, Maria Latiano, Domenica Gioffreda, Tiziana Latiano, Maria Guerra, Giuseppina Martino, Maria Rosa Valvano, Fabrizio Bossa, Francesco Perri, Anna Latiano

**Affiliations:** 1 Division of Gastroenterology and Endoscopy, Fondazione IRCCS “Casa Sollievo della Sofferenza”, Viale Cappuccini 1, 71013 San Giovanni Rotondo, Italy; 2 Quality and Accreditation Unit, Fondazione IRCCS “Casa Sollievo della Sofferenza”, 71013 San Giovanni Rotondo, Italy

**Keywords:** inflammatory bowel diseases, thiopurines, toxicity, pharmacogenetics, genotyping, *TPMT*, *NUDT15*

## Abstract

Thiopurines are employed in inflammatory bowel diseases (IBDs; Crohn’s disease, CD; ulcerative colitis, UC) to induce remission, prevent relapse, and reduce the steroid dosage, although they can sometimes be ineffective and present side effects. Genetic variations in the *TPMT* and *NUDT15* genes are well recognized to influence the therapeutic response, despite notable regional differences in their frequencies across various ethnic populations. Herein, the risk haplotypes TPMT*3A, *3B, *3C, and the variant NUDT15*3 were examined in a retrospective cohort of 383 Italian IBD patients who received azathioprine or 6-mercaptopurine. *TPMT* and *NUDT15* genotyping was performed by Sanger sequencing and TaqMan allelic discrimination, respectively. Allelic and genotype frequencies and genotype–phenotype correlations in non-responder and intolerant patients were assessed in comparison to responders. In total, 17% of patients did not respond to treatment, while 20% experienced adverse events, with leukopenia found in 13% of patients. TPMT haplotypes were found in 3.1% of patients, and 1.6% had the NUDT15*3 variant. CD patients with leukopenia had a higher frequency of the *TPMT* risk haplotype (40% vs. 4%, *p* = 0.024). Although additional validation through larger prospective studies or meta-analyses is needed, our findings support the importance of *TPMT* gene-variant assessment for forecasting azathioprine-related leukopenia in Italian IBD patients.

## 1. Introduction

Inflammatory bowel diseases (IBDs), with Crohn’s disease (CD) and ulcerative colitis (UC) as the primary forms, are persistent inflammatory conditions affecting the gastrointestinal system marked by a worldwide increasing prevalence [1,2]. Annual incidence rates reported by European studies, from 2000 to 2020, assess IBD between 10.5 and 46.14 per 100,000, with estimates for CD and UC ranging from 4.1 to 22.78 and 3.0 to 23.36 per 100,000, respectively, and a north–south gradient within individual countries, including Italy [3,4]. While CD and UC have comparable features and clinical phenotypes, including the alternation of periods of symptom exacerbation (defined as an active phase) with periods of remission (defined as an inactive phase), they vary concerning the locations of inflammation in the gastrointestinal tract and their respective immunological and histological characteristics [5].

The thiopurine compounds—including azathioprine (AZA), 6-mercaptopurine (6-MP), and thioguanine (TG)—are immunosuppressive agents belonging to the purine analog subclass of antimetabolites and represent a cornerstone in the treatment of IBDs. The thiopurines are used to maintain remission, to prevent relapses, and to reduce the steroid dosage, serving as the initial treatment option [6]. Nonetheless, 25% of patients may not respond to AZA or 6-MP, with the occurrence of widely documented thiopurine metabolism-related adverse events impacting 20–30% of patients [7,8,9]. In addition, a large interindividual variability in response to the therapy has been shown.

AZA is a pro-drug converted by the liver to 6-MP, which is metabolized through different pathways into inactive metabolites (6-TU, 6-thiouric acid; 6-MMP, 6-methylmercaptopurine) or converted into 6-thioinosine monophosphate (6-TIMP), leading to the formation of active 6-thioguanine nucleotides (6-TGNs). These active nucleotides are incorporated into cellular RNA/DNA, inhibiting cellular proliferation and inducing T-cell apoptosis by modulating Rac1 signaling [10]. The enzymes encoded by thiopurine s-methyltransferase (*TPMT*) and nudix hydrolase 15 (*NUDT15*) play a key role in the metabolic pathway of thiopurines as they modulate drug activation and limit the accumulation of toxic metabolites: TPMT can methylate 6-MP into the inactive metabolite 6-MMP or 6-TIMP, resulting in the active metabolite 6-TGNs, responsible for hepatotoxicity associated with thiopurines; NUDT15 hydrolyzes and inactivates the 6-TGNs and is associated with thiopurine-induced leukopenia.

The activity of these enzymes is influenced by genetic single-nucleotide polymorphisms (SNPs) in the *TPMT* and *NUDT15* genes, resulting in null or decreased enzyme activity. A homozygous genotype either in the *TPMT* or *NUDT15* genes reduces the enzymatic activity necessary for drug metabolism, whereas the heterozygous genotype results in decreased enzyme activity linked to heightened side effects [11]. Alterations in TPMT activity are mainly attributed to the alleles *3A (c.460G>A, rs1800460; c.719A>G, rs1142345), *3B (c.460G>A, rs1800460), and *3C (c.719A>G, rs1142345) compared to the wild-type allele (TMPT*1) [12]. About 3–14% of the general population carries the heterozygous *TPMT* genotype—which is considered an intermediate metabolizer of thiopurines—while 0.3% of the population is TPMT-deficient [13]. Although the rates of severe toxicity are comparable across various races, the occurrence of homozygotes for *TPMT* mutant alleles is approximately 0.6% in Caucasians, whereas it is very infrequent in Asians (~0.01%) [14]. Conversely, recent reports indicate that a deficiency in NUDT15 plays a more significant role in thiopurine-related toxicity among Asian populations [15]. The first recognized *NUDT15* loss-of-function polymorphism NUDT15*3 (c.415C>T, rs116855232) is frequently observed in Asians but is rare in Caucasians and Africans, with allele frequencies of 0.12 and <0.01, respectively [16]. Marked regional differences exist in the frequency of genetic variations in the *TPMT* and *NUDT15* genes among different ethnic groups and medical fields [17,18,19]. The prevalence of *TPMT* mutant alleles in the European population varies between 3.5% and 5.5%, with TPMT*3A being the most frequent variant (2–4.5%), unlike *3B and *3C (less than 1%), while homozygous variants have frequencies around 0.3% [20,21,22]. Variants of *NUDT15* in individuals of European ancestry have a prevalence of 1.3%, with the occurrence of NUDT15*3 being less than 1%, and more than 90% of patients with the variant present it in a heterozygous form while carrying the wild-type TPMT [21,23]. Regarding the Italian population, the research is limited and outdated. A study conducted in 2001 found a 5.3% frequency of defective *TPMT* alleles in 103 healthy Italian blood donors, with *3A being the predominant allele at 3.9% and *3C approaching 1%, while *3B was not present [24]. A later study in 2009 tested a large healthy Italian cohort, identifying 5.4% heterozygous variants in *TPMT*, with the frequencies of the alleles being *3A (4.3%), *3B (0.5%), and *3C (0.5%), respectively [25].

Several studies suggest that *TPMT* and *NUDT15* genotyping can tailor dosing strategies and improve treatment effectiveness while minimizing side effects in IBD patients [26,27,28]. This research emphasizes the significance of accounting for genetic differences in optimizing thiopurine therapy in order to balance therapeutic effectiveness with the mitigation of adverse effects and aid in personalized treatment, leading to improved patient clinical outcomes. Moreover, the Clinical Pharmacogenetics Implementation Consortium (CPIC) and the Dutch Pharmacogenetics Working Group (DPWG) have released clinical guidelines detailing dosing suggestions derived from *TPMT* and *NUDT15* genotypes [16,29], while the Federal Drug Administration (FDA) and the European Crohn’s and Colitis Organization (ECCO) also offer recommendations to help clinicians understand how to use genetic test findings to improve medication treatment genotypes [30,31].

Defining the strategies for customizing thiopurine dosing still remains a challenge, and advancements in medication for IBD treatment may prompt a reassessment of thiopurine use in managing IBDs. However, since the low cost and extensive accessibility of thiopurines render them a reasonable option, enhancing our comprehension of how regional and population-specific genetic diversity affects the response to thiopurines is of considerable importance. Within this framework, the purpose of our study was to evaluate the association between the NUDT15*3 variant—along with TPMT*3A, *3B, and *3C risk haplotypes—and the response and toxicity of thiopurine therapy in a cohort of Italian IBD patients. Herein, we explored the hypothesis that genetic testing for these variants could serve as a viable approach to pinpoint patients at increased risk of adverse drug reactions or a lack of responsiveness in the Italian population, thereby implementing knowledge to promote the safer and more effective use of thiopurines within this ethnic group.

## 2. Results

### 2.1. Response of Patients to AZA/5-MP Treatment

The cohort of IBD patients—consisting of a total of 383 subjects (192 CD and 191 UC)—was classified into responders, non-responders, and those intolerant to AZA/6-MP by evaluating the therapeutic efficacy and the presence of side effects throughout the treatment. The mean duration of therapy with thiopurines was 42 ± 25 months (range 1–96). The mean dosage was 145 mg/day ± 30 mg/day (range 100–200) for AZA and 75 mg/day ± 20 mg/day (range 50–125) for 6-MP. Regarding concomitant medications at the start of the thiopurine treatment, 95% of UC patients and 43% of CD patients were also receiving Mesalazine; 280 patients (73%) were concomitantly treated with steroids. Other drugs not used for IBD treatment were not considered.

Overall, 241 patients (63%) showed a response to the treatment (121 CD and 120 UC); 67 patients (17%) experienced no therapeutic effect from thiopurines (29 CD and 38 UC); and the remaining 75 patients (20%) reported therapy-related adverse events (42 CD and 33 UC). In detail, adverse events included the following: nausea and vomiting in 14 patients (19%); skin reactions in 4 patients (5%); flu-like symptoms in 5 patients (7%); infections and abdominal/thoracic pain in 13 patients (17%); acute pancreatitis in 14 patients (19%); leukopenia in 10 patients (13%); hepatic toxicity in 13 patients (17%); and 2 patients reported both acute pancreatitis and liver failure as adverse events (3%).

### 2.2. TMPT and NUDT15 Gene Polymorphisms

The genotypes for the c.460G>A and c.719A>G variants in the *TPMT* gene were successfully identified in all patients analyzed, whereas the c.415C˃T variant in the *NUDT15* gene was genotyped in 376 (188 CD and 188 UC) patients enrolled in this study (98.2%).

A total of 18 patients (12 CD and 6 UC) carried mutated alleles of SNPs in the examined genes: 12 individuals (8 CD and 4 UC) in the *TMPT* gene, and 6 individuals (4 CD and 2 UC) in the *NUDT15* gene. The frequency of allele carriers was 3.1% for the *TMPT* gene (4.2% in CD and 2.1% in UC) and 1.6% for the *NUDT15* gene (2.1% in CD and 1.1% in UC). No patients presented the mutated allele in the homozygous condition, and there were no cases of mutated alleles in both genes.

Frequencies of alleles and genotypes for the evaluated variants were consistent with the Hardy–Weinberg equilibrium (all *p*-values > 0.05). The associations between allele and genotype frequencies of SNPs in the *TMPT* and *NUDT15* genes and the response of patients to treatment with AZA/5-MP were assessed by analyzing both the entire IBD cohort and CD and UC patients individually.

The allelic and genotypic frequencies of SNPs in the *TMPT* and *NUDT15* genes revealed no statistically significant association with the response to treatment (Supplementary Tables S1 and S2). Similarly, the distribution of *TMPT* haplotype carriers did not demonstrate statistically significant differences between patients who were either non-responders or intolerant compared to those exhibiting a favorable therapeutic response (Supplementary Table S2).

However, when intolerant patients were compared to those responding to treatment based on the type of adverse event (Table 1), it was found that 29% (2/7) of patients with at least one haplotype of the *TPMT* gene experienced leukopenia, in contrast to 3% (8/244) of wild-type patients (OR = 11.8, 95%CI = 1.98–70.36, *p* = 0.027). This association persisted in the CD subgroup (40% vs. 4%, *p* = 0.024; OR = 15.7, 95%CI = 2.13–116.32).

To establish whether the presence of variants in the *TPMT* and *NUDT15* genes was associated with diseases phenotypes, we analyzed the association of genotypes with age at diagnosis, gender, risk factors (smoking, appendicitis, tonsillectomy, and family history of IBDs), disease location, disease behavior, the presence of non-perianal fistulas and perianal disease, the presence of extra-intestinal manifestations, and surgical resection. The analysis revealed no significant correlation between the genotype and clinical phenotype in patients who either did not respond to AZA/5-MP or experienced adverse toxicity events when compared to those who responded to the treatment (Supplementary Table S3).

### 2.3. Response to AZA/5-MP Treatment in Relation to Clinical–Pathological Features of IBDs

The associations between the response to thiopurine treatment and the clinical–pathological features of IBD patients were also evaluated. The variables that exhibited a statistically significant correlation are presented in Table 2 and Table 3A,B.

When examining the IBD study population (Table 2), the percentage of patients who underwent surgical resection was notably greater in the non-responder subgroup than in the responder subgroup (30% vs. 17%, *p* = 0.025). The distribution of the age at diagnosis was significantly different between patients classified as intolerant compared to those who responded to the therapy (*p* = 0.038), with a smaller percentage of patients aged 17–40 years (48% vs. 64%) and a larger percentage of patients over 40 years old (39% vs. 25%). The percentage of patients with family history of IBDs was greater between non-responders (18%) and those intolerant (20%) to treatment than the responders (6%), with *p* = 0.002 and *p* < 0.001, respectively. Furthermore, a higher numerical frequency of extra-intestinal manifestations emerged between intolerant patients compared to the responders (45% vs. 33%, *p* = 0.06).

In CD patients (Table 3A), the percentage of patients with non-perianal fistulas was higher in non-responders than responders (28% vs. 12%, *p* = 0.04), while the number of patients with a family history was significantly higher in those intolerant compared to the responders (29% vs. 8%, *p* = 0.001). Moreover, a trend toward an association between treatment resistance and the Montreal disease classification emerged, with a lower percentage of patients with inflammatory behavior and a higher percentage of those with fistulizing behavior in non-responders compared to the responders (41% vs. 59%, and 28% vs. 12%; *p* = 0.064).

In UC patients (Table 3B), the percentage of patients with a family history of IBDs was higher among the non-responders than the responders (16% vs. 3%, *p* = 0.014). Moreover, the age at diagnosis varied among UC patients who showed intolerance to AZA/5-MP and those who benefited from treatment (*p* = 0.035), revealing a smaller proportion of patients aged 17–40 years (42% vs. 60%) and a greater percentage of patients over 40 years (55% vs. 31%). Furthermore, the percentage of patients without a smoking habit was higher among those intolerant than the responders (70% vs. 49%, *p* = 0.037).

## 3. Discussion

Thiopurines are widely used for maintaining remission or reducing steroid use in patients with IBDs, and they are currently also recommended in association with infliximab [31,32]. Nonetheless, many patients show insufficient responses to treatment or experience adverse events, situations that require dose modifications or therapy discontinuation. Myelotoxicity is one of the most frequent reasons for drug withdrawal, and genetic polymorphisms in the *TPMT* and *NUDT15* genes are well recognized as causing leukopenia. While haplotypes of the *TPMT* gene play a major role in the Western population, *NUDT15* variants are thought to be more relevant to Asians and South Asians [16]. Evaluating *TPMT* and *NUDT15* pharmacogenomics is advantageous for tailoring the decrease in thiopurine-associated toxicity, and it can also affect pediatric care, where choices regarding dose modification or the selection of an alternative therapy hold considerable importance in clinical practice [33,34].

Different clinical and scientific bodies have suggested guidelines that detail thiopurine dosing recommendations according to the *TPMT* and *NUDT15* genotypes, along with suggestions regarding genetic testing [16,29,30,31]. Furthermore, a pragmatic controlled trial was carried out to assess the cost-effectiveness of a *TPMT* genotyping test for guiding AZA prescriptions, randomizing patients with gastroenterological or rheumatological conditions—including CD and UC—to receive *TPMT* genotyping before AZA or current practice. This prospective economic evaluation revealed that *TPMT* genotyping led to moderately lower costs than current practice, albeit with a mean incremental quality-adjusted life-years close to zero between the two study arms [35]. A recent study also assessed the cost-effectiveness of pretreatment *NUDT15* pharmacogenetic testing in IBD patients, indicating that a combined *TPMT* and *NUDT15* analysis is the most cost-effective option among European participants and recommended to consider reflex *NUDT15* testing for patients exhibiting reduced TPMT activity or the loss-of-function genotype [23]. In addition, these authors indicated that the *NUDT15* pharmacogenetic test was cost-effective and enhanced the safety of thiopurine dosing by decreasing the frequency and cost of thiopurine blood monitoring for patients without *TPMT* and *NUDT15* risk variants.

In the current study, 17% of all IBD patients exhibited no clinical response to the treatment with AZA/5-MP, and 20% experienced adverse events linked to the use of the thiopurines, with leukopenia identified in 13% of patients—in line with evidence derived from the literature [10,36]. Twelve patients (3.1%) carried heterozygous variants in the *TPMT* gene, while six patients (1.6%) presented the NUDT15*3 variant. Furthermore, no patient showed variants in both the *TPMT* and *NUDT15* genes. The low frequency of *TPMT* and *NUDT15* variant carriers found in our study aligns with previously reported research, thereby indicating the actual low prevalence of variants in these genes within both the European [20,21,23] and Italian populations [24,25].

Allelic and genotypic frequencies of the *TMPT* and *NUDT15* SNPs showed no statistically significant correlation with the treatment response, and the distribution of *TMPT* haplotypes was similar between non-responder patients and those experiencing adverse events compared to the responders; however, when the distribution of *TPMT* haplotypes and NUDT15*3 variant carriers was examined in relation to the types of adverse events in intolerant patients versus those who achieved benefit from treatment, leukopenia was observed more often in CD patients with at least one haplotype of the *TPMT* gene.

In an era when treatment options are constantly growing and the personalized medicine model is gaining traction, the capacity to foresee the effects and the potential adverse reactions of thiopurines and to tailor treatment appropriately via drug selection or dose adjustment according to the genetic profile of each individual patient represents the purpose and future application of these medications [26]. In this scenario, the genetic variations in the *TPMT* and *NUDT15* genes are recognized to affect the safety of thiopurine treatment and are mainly linked to the risk of myelosuppression related to the therapy [37]. The low frequency of recognized mutations has limited the detection of meaningful associations with thiopurine treatment response and significant phenotype–genotype correlations; however, the findings in this study emphasize the necessity of assessing the variants in the *TPMT* gene, establishing it as a standard of care for predicting AZA-related leukopenia. Further research is needed to find out whether *TPMT* genetic polymorphisms are responsible for leukopenia in CD patients and whether thiopurines undergo different enzymatic modifications giving rise to active and inactive metabolites that influence their therapeutic effects differently in IBD patients.

With respect to clinical features associated with IBDs, we observed a higher percentage of patients who underwent surgical resection among the non-responders to the treatment, as well as a higher percentage of patients aged over 40 years at diagnosis among those classified as intolerant. The association between the age at diagnosis and the intolerance to AZA/5-MP persisted among the subgroup of UC patients, where a greater count of non-smokers compared to those who responded to thiopurines was also observed. Furthermore, a family history of IBDs occurred more often among both the UC patients classified as non-responders and the CD patients classified as intolerant when compared to those showing a clinical response to thiopurines.

The correlation between toxicity of thiopurines and a higher risk of surgical resection has been described in UC patients and, although with a milder impact, in CD patients as well [38]. Overall, it should be noted that when medical treatment fails, surgery is inevitably the therapeutic option for treatment of IBD patients. Clinical predictors, such as age and sex, a smoking habit, and genetic factors, can influence the likelihood of experiencing adverse events, impacting treatment decisions and patient outcomes. In particular, women over 40 years of age at the start of thiopurine treatment are at greater risk of treatment-related side effects and drug discontinuation than younger women and men of all ages [39]. Moreover, older individuals with IBDs may have a higher likelihood of experiencing complex comorbidities and/or be on other medications, which can influence their tolerance to treatments [40]. A smoking habit, even if not consistently linked to thiopurine-related adverse events, can influence the overall disease course and severity. Smoking elevates the risk of onset and exacerbates the progression of CD, while it offers protection against the onset and reduces the severity of UC [41,42]. In addition, several studies have demonstrated the association between smoking and increased levels of 6-thioguanine nucleotides [43,44]. While a positive family history of IBDs is a significant risk factor for developing IBDs, and can also influence disease characteristics and treatment response [45], no association has currently been identified between family history of IBDs and patients’ response to thiopurines. Therefore, the epidemiological data observed in this study cannot be clearly interpreted since the genetic factors possibly related to family history might contribute to the genetic background, which is linked to thiopurine metabolism. Finally, while further research on clinical predictors is needed to refine the ability to predict thiopurine-related adverse events in IBD patients, by taking these clinical and genetic factors into account, healthcare providers can make more informed decisions about therapy, potentially improving treatment outcomes and minimizing adverse events.

Based on the data obtained, it is necessary to make some observations on our research since several factors are important when assessing how IBD patients respond to thiopurine treatment. Indeed, variations in the *TPMT* gene do not always explain the differences in enzyme activity among individuals [46,47]. New gene variants may also affect TPMT activity, along with genetic variations in non-coding regions [46,48,49,50]. Furthermore, genes for co-factors regulating TPMT activity and interactions with other drugs could lead to side effects [51,52]. Additionally, other genes linked to thiopurine toxicity have been identified (*ITPA*, *HPRT*, *XDH*, *GSTM1*, *XDH*, and *GMPS*) [53], and recent studies suggest that gut microbiota may influence treatment effectiveness and disease remission, though it remains unclear if it affects the toxicity of thiopurines [54]. Furthermore, apart from gene polymorphism genotyping, measuring the levels of thiopurine metabolites, 6-TGN and 6-MMP, in hemolysates is a common method utilized to enhance thiopurine drug treatment [55]. A therapeutic window has been established for these metabolites to aid clinicians in balancing therapeutic effectiveness, adjusting thiopurine medication dosages to achieve optimal therapeutic levels while reducing the risk of side effects, and identifying non-adherent or underdosed patients. In summary, 6-TGN levels between 235 and 450 pmol/8 × 10^8^ red blood cells typically correlate with improved clinical response and decreased disease activity in IBDs. Levels below 235 pmol/8 × 10^8^ red blood cells suggest non-responsiveness to treatment or hint at non-compliance, whereas excessively high levels may elevate the risk of leukopenia. Likewise, 6-MMP concentrations exceeding 5700 pmol/8 × 10^8^ red blood cells might suggest an elevated risk of hepatotoxicity. Furthermore, when a patient does not show improvement with thiopurine therapy despite proper dosing, testing for metabolites can assist in identifying whether the unresponsiveness is dependent on inadequate absorption, quick conversion to inactive metabolites, or other factors. Nonetheless, the application of metabolite testing in clinical practice might still be constrained by aspects like expense and limited access to specialized laboratories, and discrepancies in laboratory techniques can influence results.

We acknowledge several limitations in our findings. The prevalence of identified mutations in our study group—especially the very low frequency noted for the NUDT15*3 variant and the limited number of meaningful associations found—constrains the potential to make definitive conclusions regarding the relevance of *TMTP* and *NUDT15* testing in our Italian population of IBD patients. Although not unexpected according to the literature [22], we did not identify homozygous variants in the genes examined nor patients with variants in both genes. Finally, we acknowledge the retrospective nature of our study.

In conclusion, despite these considerations, we have shown that genetic variations in the *TPMT* gene could be useful for predicting thiopurine-associated leukopenia in Italian patients undergoing treatment. To reinforce the role of thiopurines in IBD therapy and improve their efficacy, it is notable to recognize the necessity for additional validation of our findings through larger prospective studies or meta-analyses, and it is advisable to adopt a strategy that comprehensively addresses the pharmacogenetic factors associated with these compounds.

## 4. Materials and Methods

### 4.1. Patients Enrolled in This Study

A total of 383 IBD patients (228 males, mean age at diagnosis: 33 ± 14 years) receiving AZA/6-MP treatment at the Division of Gastroenterology and Endoscopy, Fondazione IRCCS “Casa Sollievo della Sofferenza” Hospital, San Giovanni Rotondo, were retrospectively enrolled in this study. In detail, 192 were CD patients (116 males, mean age at diagnosis: 29 ± 13 years) and 191 were UC patients (112 males, mean age at diagnosis: 37 ± 15 years). For each patient, diagnosis of CD and UC was established by clinical, endoscopic, histological, and radiological criteria in accordance with guidelines published by ECCO [56]. The Montreal Classification was utilized to evaluate the location and behavior of both CD and UC [57,58]. The demographics and comprehensive phenotypic information of patients can be found in Table 4.

Patients were assessed and classified through a careful examination of medical records as responder, non-responder, and intolerant to treatment with a thiopurine drug [59]. Briefly, the evaluation of response to the therapy was considered adequate after six months of treatment (2–2.5 mg/kg and 1–1.5 mg/kg for AZA and 6-MP, respectively). Response to therapy was assessed using clinical indices (HBI < 5 and PMS < 2) and at least one objective marker of disease activity (CRP level < 5 mg/dL; fecal calprotectin level < 150 μg/g; reduction of at least 3 points in SES-CD or ≥1 point in endoscopic Mayo score; or reduction of at least 2 mm in bowel thickness as assessed by bowel ultrasound or small-bowel MR enterography). Responder patients were able to achieve or maintain disease remission by suspending steroids treatment; non-responders required drug withdrawal due to its ineffectiveness and an inability to manage steroid discontinuation; and those classified as intolerant needed to interrupt therapy or reduce the dosage due to adverse effects. Both dose-dependent (myelosuppression, rare bacterial infections, and hepatic toxicity) and idiosyncratic (flu-like symptoms, fever, rash, arthralgia, and pancreatitis or gastrointestinal disorders) side effects were considered [60]. Bone marrow toxicity was defined as having either leukopenia (white blood count < 3.0 × 10^9^/L) and/or thrombocytopenia (platelet count < 100.00 × 10^6^/L) that improves following treatment continuation or dose adjustment [61]. Hepatotoxicity was characterized by a serum alanine transaminase increase exceeding twice the upper normal limit and resolution following drug withdrawal or dosage reduction. Pancreatitis was characterized by severe epigastric pain and nausea accompanied by serum amylase and lipase levels exceeding twice the normal upper threshold.

This study was conducted in accordance with the Declaration of Helsinki and approved by the ethical committee of the Fondazione IRCCS “Casa Sollievo della Sofferenza” Hospital (Prot. N.12701/08, dated 26 September 2007). Written informed consent was obtained prior to blood collection from patients and from legal guardians for subjects under 18 years of age.

### 4.2. DNA Isolation and TPMT and NUDT15 Genotyping

Genomic DNA was obtained from peripheral blood samples using the QIAmp DNA Blood Maxi Kit (Qiagen, Hilden, Germany), according to the guidelines provided by the manufacturer.

The c.460G>A and c.719A>G SNPs in the *TPMT* gene were identified as previously detailed [62] through DNA sequence amplification using the polymerase chain reaction with specific oligonucleotide primer sets, followed by a DNA sequencing analysis on an ABI 3500 Dx DNA sequencer (Applied Biosystems, Foster City, CA, USA). The c.415C>T polymorphism in the *NUDT15* gene was genotyped through allelic discrimination on an ABI PRISM 7900 Sequence Detection System (Applied Biosystems, Foster City, CA, USA) using the TaqMan SNP Genotyping Assay (Cat. No. 4351379; Assay ID: c_154823200_10) (Applied Biosystems, Foster City, CA, USA) according to the manufacturer’s guidelines.

### 4.3. Statistical Analysis

To assess the impact of *TPMT* and *NUDT15* variants on the efficacy and side effects of thiopurines, we analyzed the influence of the SNPs in a case-control framework in which the non-responders and the intolerant patients were designated as cases and the responders as controls. Hardy–Weinberg equilibrium tests were performed for all the investigated SNPs. Using the software PS Power and Sample Size v.2.1.31, given the study sample size and a minor allele frequency of 1% in the control population, we had 65% power at the 5% significance level to detect a difference of +/− 5% in allele frequency in the case group. For case-control analysis, the comparisons of genotypes and allelic frequencies were performed using the chi-square test or Fisher’s exact test, where appropriate. Baseline characteristics were assessed with standard descriptive statistics. Genotype–phenotype associations were analyzed by univariate analysis and expressed as the Odds Ratio (OR) with 95% confidence intervals (95% CIs). All analyses were performed using the SPP v.13 software package (Chicago, IL, USA). The *p*-values < 0.05 were considered statistically significant.

## Figures and Tables

**Table 1:** Distribution of *TPMT* haplotypes and NUDT15*3 variant carriers based on the type of adverse event in patients with inflammatory bowel diseases (IBDs) who are intolerant versus those who respond to AZA/5-MP.

	TPMT*3A, *3B, *3C			NUDT15*3 ^$^		
	Mutant	Wild-Type	*p*-Value	Mutant	Wild-Type	*p*-Value
Hepatotoxicity (*n* = 15)	1 (17)	14 (6)	ns	0 (0)	14 (6)	ns
Acute pancreatitis (*n* = 16)	0 (0)	16 (6)	ns	0 (0)	16 (6)	ns
Leukopenia (*n* = 10)	2 (29)	8 (3)	0.027	1 (33)	9 (4)	ns
Nausea/vomiting (*n* = 14)	0 (0)	14 (6)	ns	1 (33)	13 (5)	ns
Flu-like symptoms (*n* = 5)	0 (0)	5 (2)	ns	0 (0)	5 (2)	ns
Skin reaction (*n* = 4)	0 (0)	4 (2)	ns	0 (0)	4 (2)	ns
Infections/pain (*n* = 13)	1 (17)	12 (5)	ns	0 (0)	13 (5)	ns

^$^: the NUDT15*3 allelic variant is not available for 1 intolerant patient.; ns: not statistically significant (*p* > 0.05)

**Table 2:** Associations between response to AZA/5-MP and clinical–pathological variables of patients with inflammatory bowel diseases (IBDs).

	Responder	Non-Responder	Intolerant		
	(R)	(NR)	(I)	*p*-Value	
	*n* = 241	*n* = 67	*n* = 75	NR vs. R	I vs. R
Resection, *n* (%)					
No	199 (83)	47 (70)	57 (76)	0.025	
Yes	42 (17)	20 (30)	18 (24)		
Age at diagnosis *, *n* (%)					
A1: ≤16	28 (12)	14 (21)	10 (13)		0.038
A2: 17–40	153 (64)	36 (54)	36 (48)		
A3: >40	59 (25)	17 (25)	29 (39)		
Family history of IBDs *, *n* (%)					
No	223 (94)	55 (82)	59 (80)	0.002	<0.001
Yes	14 (6)	12 (18)	15 (20)		
EIMs, *n* (%)					
No	161 (67)	52 (78)	41 (55)	0.09	0.06
Yes	80 (33)	15 (22)	34 (45)		

EIMs: extra-intestinal manifestations. * Data not available for some patients (*n* = 1, age at diagnosis; *n* = 5, family history of IBDs).

**Table 3:** (**A**) Association between response to AZA/5-MP and clinical–pathological variables in patients with Crohn’s disease (CD). (**B**) Association between response to treatment and clinical–pathological variables in patients with ulcerative colitis (UC).

**(A)**					
	**Responder**	**Non-Responder**	**Intolerant**		
	**(R)**	**(NR)**	**(I)**	***p*-Value**	
	***n* = 121**	***n* = 29**	***n* = 42**	**NR vs. R**	**I vs. R**
Non-perianal fistula, *n* (%)					
No	107 (88)	21 (72)	21 (72)	0.04	
Yes	14 (12)	8 (28)	8 (28)		
Family history of IBDs *, *n* (%)					
No	109 (92)	23 (79)	30 (71)		0.001
Yes	10 (8)	6 (21)	12 (29)		
Behavior CD, *n* (%)					
B1: Inflammatory	72 (59)	12 (41)	21 (50)	0.064	
B2: Stenosing	35 (29)	9 (31)	15 (36)		
B3: Fistulizing	14 (12)	8 (28)	6 (14)		
**(B)**					
	**Responder**	**Non-Responder**	**Intolerant**		
	**(R)**	**(NR)**	**(I)**	***p*-Value**	
	***n* = 120**	***n* = 38**	***n* = 33**	**NR vs. R**	**I vs. R**
Family history of IBDs *, *n* (%)					
No	114 (97)	32 (84)	29 (91)	0.014	
Yes	4 (3)	6 (16)	3 (9)		
Age at diagnosis, *n* (%)					
A1: <16 A2: 17–40 A3: >40	11 (9)	6 (16)	1 (3)		0.035
	72 (60)	19 (50)	14 (42)		
	37 (31)	13 (34)	18 (55)		
Smoking habit *, *n* (%)					
No	55 (49)	25 (68)	23 (70)		0.037
Yes	57 (51)	12 (32)	10 (30)		

* Data not available for 2 patients.

**Table 4:** Clinical and demographic traits of individuals with inflammatory bowel diseases (IBDs), Crohn’s disease (CD) and ulcerative colitis (UC), enrolled in this study.

	IBDs *n* = 383	CD *n* = 192	UC *n* = 191
Age at diagnosis *, mean ± DS	33 ± 14	29 ± 13	37 ± 15
A1: ≤16, *n* (%)	52 (14)	34 (18)	18 (9)
A2: 17–40, *n* (%)	225 (59)	120 (63)	105 (55)
A3: >40, *n* (%)	105 (27)	37 (19)	68 (36)
Gender, M/F (%M)	228/155 (60)	116/76 (60)	112/79 (59)
Smoking habit *, *n* (%)			
No	192 (52)	89 (47)	103 (57)
Yes	180 (48)	101 (53)	79 (43)
Appendicitis *, *n* (%)			
No	318 (87)	150 (80)	168 (94)
Yes	48 (13)	38 (20)	10 (6)
Tonsillectomy *, *n* (%)			
No	306 (86)	163 (90)	143 (82)
Yes	50 (14)	19 (10)	31 (18)
Family history of IBDs *, *n* (%)			
No	337 (89)	162 (85)	185 (93)
Yes	41 (11)	28 (15)	13 (7)
Localization UC, *n* (%)			
E1: Rectum			3 (2)
E2: Colon sx			88 (46)
E3: Pancolitis			100 (52)
Localization CD, *n* (%)			
Ileum		83 (43)	
Ileum–colon		80 (42)	
Colon		24 (12)	
Upper GI tract		5 (3)	
Behavior CD, *n* (%)			
B1: Inflammatory		105 (54.7)	
B2: Stenosing		59 (30.7)	
B3: Fistulizing		28 (14.6)	
Perianal disease, yes/no (%)	43/340 (11)	37/155 (19)	6/185 (3)
Non-perianal fistulas, yes/no (%)	28/355 (7)	28/164 (15)	0/191 (0)
Resection, yes/no (%)	80/303 (21)	65/127 (34)	15/176 (8)
EIMs, yes/no (%)	129/254 (34)	74/118 (39)	55/136 (29)

GI: gastrointestinal; EIMs: extra-intestinal manifestations. * Data not available for some patients (*n* = 1, age at diagnosis; *n* = 11, smoking habit; *n* = 17, appendicitis; *n* = 27, tonsillectomy; *n* = 5, family history of IBDs).

## Data Availability

The original contributions presented in this study are included in the article/Supplementary Materials. Further inquiries can be directed to the corresponding author.

## Supplementary Materials

The following supporting information can be downloaded at https://www.mdpi.com/article/10.3390/ijms26167860/s1.

## Abbreviations

The following abbreviations are used in this manuscript:

6-MMP	6-Methylmercaptopurine
6-MP	6-Mercaptopurine
6-TC	6-Thiouric acid
6-TGNs	6-Thioguanine nucleotides
6-TIMP	6-Thiosine monophosphate
AZA	Azathioprine
CD	Crohn’s disease
CI	Confidence interval
CPIC	Clinical Pharmacogenetics Implementation Consortium
CRP	C-reactive protein
DPWG	Dutch Pharmacogenetics Working Group
ECCO	European Crohn’s and Colitis Organization
EIM	Extra-intestinal manifestation
FDA	Federal Drug Administration
GI	Gastrointestinal
HBI	Harvey–Bradshaw Index
IBD	Inflammatory bowel disease
MR	Magnetic resonance
NUDT15	Nudix hydrolase 15
OR	Odds Ratio
PMS	Partial Mayo score
SES-CD	Simple Endoscopic Score for Crohn’s Disease
SNP	Single-nucleotide polymorphisms
TG	Thioguanine
TPMT	Thiopurine s-methyltransferase
UC	Ulcerative colitis
